# Carnosic acid in topical rosemary extract enhances skin repair via TRPA1 activation

**DOI:** 10.1172/jci.insight.196267

**Published:** 2025-10-23

**Authors:** Emmanuel Rapp, Jiayi Pang, Borna Saeednia, Stephen Marsh Prouty, Christopher A. Reilly, Thomas H. Leung

**Affiliations:** 1 Department of Dermatology and; 2 Department of Chemistry, University of Pennsylvania, Philadelphia, Pennsylvania, USA.; 3 Department of Pharmacology and Toxicology, Center for Human Toxicology, University of Utah, Salt Lake City, Utah, USA.; 4 Corporal Michael J. Crescenz Department of Veterans Affairs Medical Center, Philadelphia, Pennsylvania, USA.

**Keywords:** Dermatology, Inflammation, Skin, Therapeutics

## Abstract

Mammalian skin wounds typically heal with a scar, characterized by fibrotic tissue that disrupts original tissue architecture and function. Therapies that limit fibrosis and promote regenerative healing remain a major unmet clinical need. Rosemary extract, particularly in the form of topical oils and creams, has gained widespread public attention for its purported wound-healing properties. However, its efficacy and mechanism of action remain poorly understood. We show in adult wound healing mouse models that an ethanol-based rosemary extract accelerates the speed of wound healing and mitigates fibrosis. Mechanistically, we identify that carnosic acid, a major bioactive component of rosemary leaves, activates the transient receptor potential ankyrin 1 (TRPA1) nociceptor on cutaneous sensory neurons to enhance tissue regeneration. Mice lacking TRPA1 in sensory neurons do not exhibit these pro-regenerative responses, confirming its role as a critical mediator. Together, these findings suggest that topical rosemary extract may represent an effective and accessible therapeutic approach to improve skin repair outcomes.

## Abstract 2

Carnosic acid in topical rosemary extracts reduce scar formation in injured skin.

## Graphical abstract

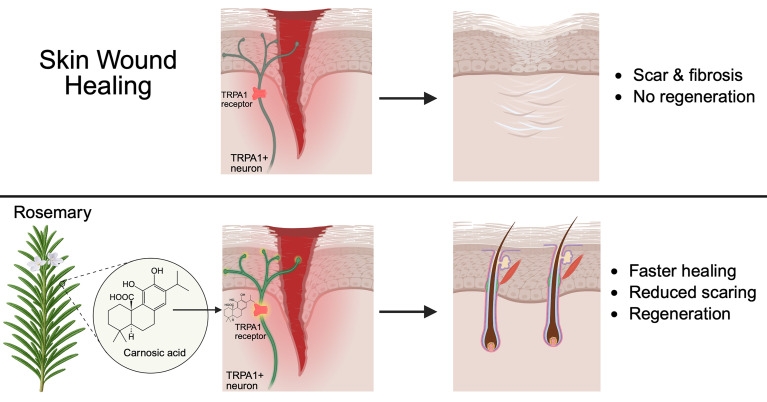

## Introduction

Skin wounds affect 100 million patients per year in the United States, and these wounds may heal by 2 biological processes: scar formation or tissue regeneration (1, 2). Scars cause loss of original tissue architecture and function, while tissue regeneration reconstitutes the major components of the skin, such as hair follicles, sebaceous glands, and cartilage (3). Most human skin wounds heal with a fibrotic scar, which, depending on the location, may cause substantial functional morbidity. Methods to prevent scar formation or to promote scarless wound healing remain a major unmet clinical need (4).

Ear hole closure is a well-established mouse model of skin injury (5–8). Typically, 2 mm through-and-through holes in the ear pinna of wild-type (WT) C57BL/6J mice heal with a scar and remain open, similar to a human earring piercing. We and others have previously shown that some strains of mice may heal with tissue regeneration that results in the complete closure of ear holes, and the healed tissue exhibits return of original tissue architecture and decreased fibrosis. More specifically, we previously showed that activation of transient receptor potential ankyrin 1 (TRPA1) nociceptor by imiquimod (IMQ) or allyl isothiocyanate (AITC) promotes scarless wound healing (9). Activated TRPA1 stimulated local production of IL-23 by dermal dendritic cells, leading to activation of circulating dermal IL-17– and IL-22–producing γδ T cells, promoting full ear wound closure.

Herbs and plants have historically been used as successful disease treatments due to their medicinal properties, and approximately 25% of drugs prescribed worldwide are of plant origin (10, 11). Social media platforms, including TikTok, Instagram, and YouTube, have numerous videos describing the potential therapeutic effects of the *Rosmarinus officinalis* (rosemary) plant to reduce scar formation, promote hair growth, and improve facial skin health, with some of the most popular videos amassing over 20 million views (12–14). The rosemary-containing health care products market was valued at USD $980.8 million in 2023 (15). Despite the widespread promulgation and belief about the medicinal properties of rosemary, there is a lack of robust and rigorous scientific studies detailing its therapeutic effects.

We demonstrate that rosemary extract promotes scarless wound healing in mouse skin and identify carnosic acid as the active ingredient. We show that rosemary activates the TRPA1 receptor on sensory nerves to promote tissue regeneration.

## Results

### Rosemary promotes scarless wound healing.

**Figure 1:**
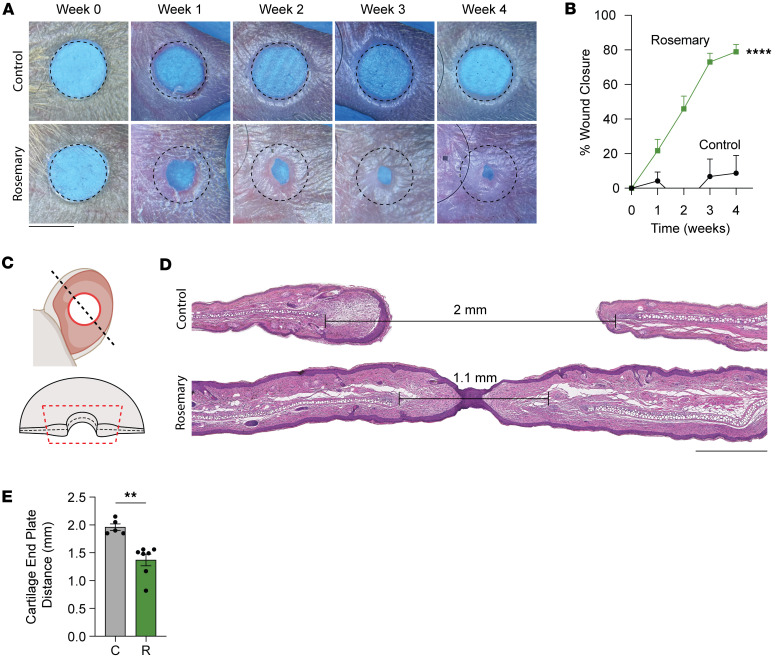
Topical rosemary cream promotes cutaneous mouse ear wound regeneration. (**A**) Representative photographs of vehicle control cream- and rosemary cream-treated WT ears. Dotted circle represents original 2 mm hole punch size. Black scale bar represents 2 mm. (**B**) Percentage of wound closure in vehicle control cream- (solid black line, *n* = 7) and rosemary cream-treated (solid green line, *n* = 12) WT mouse ears. Two-way ANOVA. (**C**) Schematic of mouse ear histology. (**D**) H&E-stained representative tissue sections from control- and rosemary cream-treated WT mouse ears. Distance between opposing cartilage endplates is indicated. Black scale bar represents 0.5 cm. (**E**) Bar plot quantifying cartilage endplate distance between control cream- and rosemary cream-treated WT mouse ears (C = control, R = rosemary). Student’s *t* test 2-tailed and unpaired. Data are presented as mean ± SEM. **P < 0.01, ****P < 0.0001.

**Figure 2:**
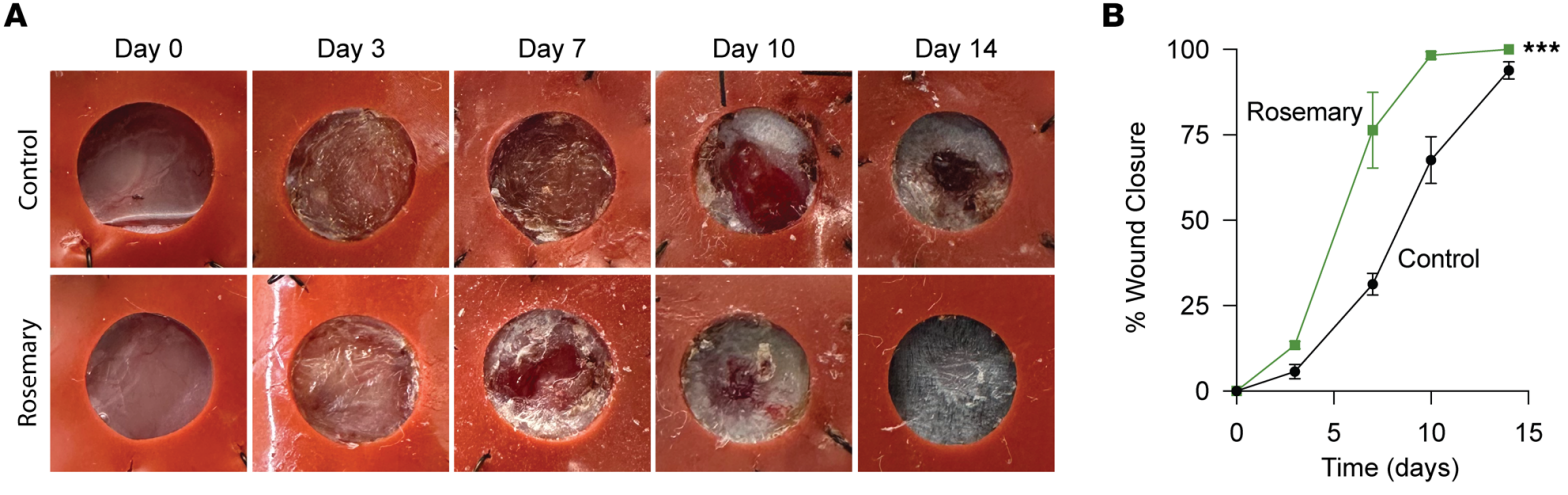
Topical rosemary cream promotes cutaneous mouse back wound closure. (**A**) Representative photographs of vehicle control cream- and rosemary cream-treated stented dorsal back wounds on WT mice. Stent hole diameter is 6 mm. (**B**) Percentage of wound closure in vehicle control cream- (solid black line, *n* = 5) and rosemary cream-treated (solid green line, *n* = 4) WT mouse dorsal stented back wounds. Two-way ANOVA. ***P < 0.001. Data are presented as mean ± SEM.

To test rosemary’s role in wound healing, we purchased rosemary from the supermarket, physically minced the leaves, performed an ethanol extraction, and spatulated the mixture into a Cetaphil cream base. After performing a 2 mm through-and-through ear punch through the pinna of mouse ears, we applied rosemary or vehicle control cream on the ears daily for 4 weeks. Rosemary cream closed ear holes to a smaller size compared with vehicle control cream-treated ears (Figure 1, A and B). Histological analysis of rosemary cream-treated mouse ear wounds exhibited increased return of normal tissue architecture, with regeneration of hair follicles, sebaceous glands, and subcutaneous fat. We also observed shortened distances between opposing cartilage end plates, supporting cartilage regeneration (Figure 1, C–E). Mouse ears are a unique anatomic location, and we wanted to determine whether these observations were generalizable to other areas of skin. Stented dorsal back skin wounds on mice typically heal with scar formation but may also exhibit regenerative phenotypes (16). Stented 6 mm dorsal back skin wounds treated with rosemary cream displayed faster reepithelialization than control mice (Figure 2, A and B). This suggests that rosemary treatment can promote wound healing at other skin sites. Together, these data show that a cream containing ethanol-extracted rosemary leaves promotes mouse wound regeneration. Next, we wanted to identify the active ingredient.

### Carnosic acid in rosemary leaves promotes regeneration.

**Figure 3:**
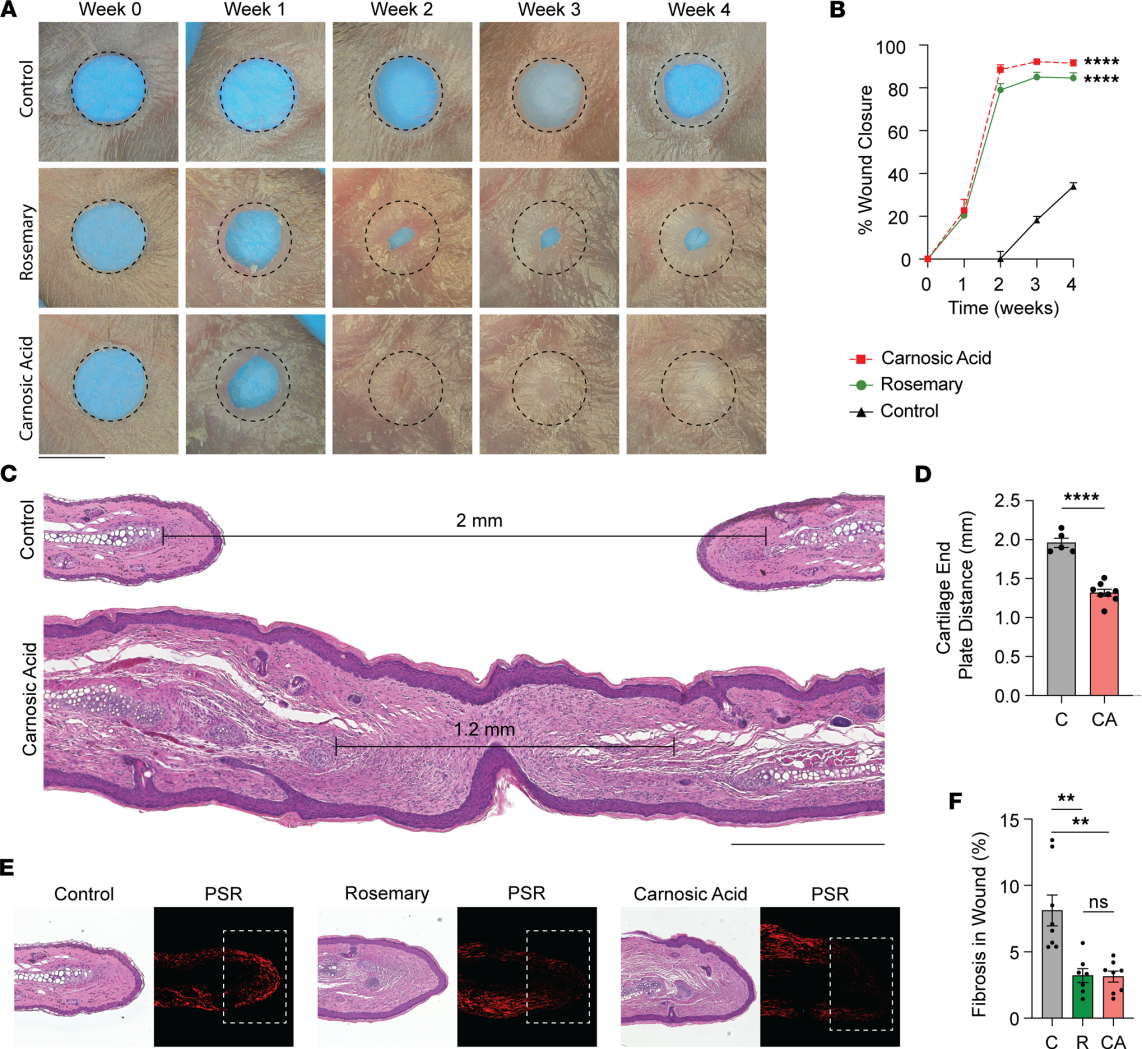
Topical carnosic acid cream promotes cutaneous mouse ear wound regeneration. (**A**) Representative photographs of vehicle control cream-, rosemary cream-, or carnosic acid cream-treated WT ears. Dotted circle represents original 2 mm hole punch size. Black scale bar represents 2 mm. (**B**) Percentage of wound closure in vehicle control cream- (solid black line, *n* = 12), rosemary cream-treated (solid green line, *n* = 12), or carnosic acid cream-treated (dotted red line, *n* = 14) WT mouse ears. Two-way ANOVA. (**C**) H&E-stained representative tissue sections from vehicle control cream- and carnosic acid cream-treated WT mouse ears. Distance between opposing cartilage endplates is marked. Black scale bar represents 0.5 cm. (**D**) Bar plot quantifying cartilage endplate distance in between control- and carnosic acid cream-treated WT mouse ears (C = control, CA = carnosic acid). Student’s *t* test 2-tailed and unpaired. (**E**) Representative H&E-stained or PSR-stained images of WT mouse ears treated with vehicle control cream, rosemary cream, or carnosic acid cream. (**F**) Bar plot quantifying fibrosis by PSR stain at the wound edge tissue from vehicle control cream-, rosemary cream-, or carnosic acid cream-treated WT mouse ears (C = control, R = rosemary, CA = carnosic acid). Student’s *t* test 2-tailed and unpaired. Data are presented as mean ± SEM. **P < 0.01, ****P < 0.0001.

Although different rosemary-containing extracts have undergone mass spectrometry profiling, the chemical composition resulting from our ethanol-based extraction method remained unknown (17). We performed ultraperformance liquid chromatography (UPLC) coupled with mass spectrometry on our ethanol-based rosemary extract. Consistent with prior studies, the 2 most abundant compounds were carnosic acid and carnosol, its oxidation product (Table 1) (18, 19). To directly test if carnosic acid may enhance wound healing, we compounded carnosic acid (5 mg/mL) into a Cetaphil cream base and performed our ear hole closure assay. After 4 weeks, carnosic acid cream-treated ears closed to an average of about 90% of the original wound size compared with about 35% for vehicle control cream-treated ears (Figure 3, A and B). Histological analysis of carnosic acid cream-treated mouse ear wounds stained with H&E revealed increased return of hair follicles, sebaceous glands, and subcutaneous fat. A shorter distance between the opposing cartilage end plates supported cartilage regeneration (Figure 3, C and D). Finally, we measured fibrosis by Picrosirius red (PSR) stain. Ears treated with either rosemary or carnosic acid cream revealed significantly less fibrosis at the wound edge compared with vehicle control cream (Figure 3, E and F). Taken together, carnosic acid is a key biologically active compound in the rosemary plant that promotes scarless wound healing. We believe its effectiveness and potency are similar to rosemary cream, with only the week 2 time point being statistically significant between the 2 treatments (*P* = 0.0129).

### Rosemary extract activates the TRPA1 ion channel.

**Figure 4:**
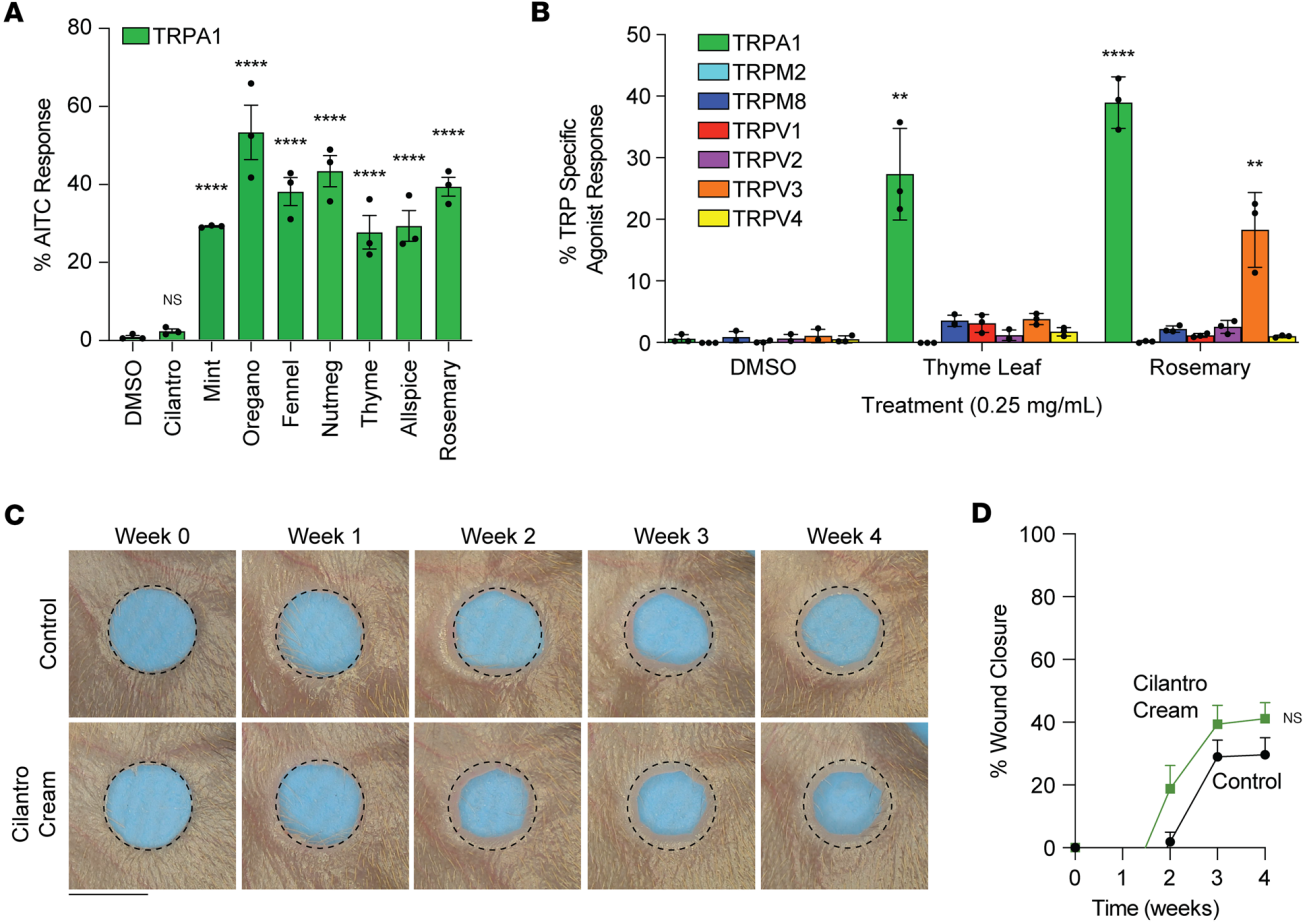
Rosemary extract activates the TRPA1 nociceptor. (**A**) Calcium flux assay responses of TRPA1 overexpressing HEK293 cells treated with different extracts reported as a percentage of a TRPA1-specific agonist. One-way ANOVA. (**B**) Calcium flux assay responses of HEK293 cells stably overexpressing different TRP ion channels treated with rosemary or thyme extract reported as percentage of each TRP channel’s specific agonist. Student’s *t* test 2-tailed and unpaired between DMSO response and thyme or rosemary response. (**C**) Representative photographs of vehicle control cream- or cilantro cream-treated WT ears. Dotted circle represents original 2 mm hole punch size. Black scale bar represents 2 mm. (**D**) Percentage of wound closure in vehicle control cream- (solid black line, *n* = 6) or cilantro cream-treated (solid green line, *n* = 7) WT mouse ears. Two-way ANOVA. Data are presented as mean ± SEM. **P < 0.01, ****P < 0.0001.

Prior work demonstrated that carnosic acid acts as a ligand for the TRPA1 receptor, and our lab showed that the activation of TRPA1 promotes full ear wound closure in mice (9, 20). To test if rosemary-induced ear wound closure was mediated by TRPA1, we screened whether rosemary and multiple other plant extracts may act as agonists of the TRPA1 channel. Compared with AITC as a positive control, we found that mint, oregano, fennel seed, nutmeg, thyme, allspice, and rosemary induced TRPA1 receptor-mediated calcium influx (Figure 4A and Supplemental Figure 1; supplemental material available online with this article; https://doi.org/10.1172/jci.insight.196267DS1). We next compared whether thyme and rosemary extracts activated other members of the TRP ion channel family. Both rosemary and thyme extracts specifically activated the TRPA1 ion channel, although rosemary modestly activated the TRPV3 channel (~15%) (Figure 4B). We noted that cilantro did not activate TRPA1 (Figure 4A), and cilantro has not been reported to generate carnosic acid (21–23). As a negative control, we compounded an ethanol-based cilantro extract into a Cetaphil cream base and repeated our ear hole assay. Cilantro cream did not result in increased ear hole closure compared with vehicle control cream-treated mouse ears (Figure 4, C and D).

Based on our prior work, we showed that local activation of TRPA1 with IMQ or AITC improved wound healing on distal sites, suggesting a systemic effect. We tested this possibility in 2 ways. (a) Wound-induced hair neogenesis (WIHN) involves large excisional back wounds that may regenerate with new hair follicles (24). We performed WIHN on WT mice and rubbed rosemary cream or control cream on the ears of mice every day for 28 days. Compared with control mice, rosemary-treated mice did not exhibit more hair follicle regeneration (Supplemental Figure 2, A and B). (b) We rubbed control cream or rosemary cream on shaved dorsal back skin and performed our ear hole closure assay. We observed a minimal, not clinically relevant, difference in ear hole closure between rosemary-treated mice and control mice (Supplemental Figure 2, C and D). We conclude that rosemary cream only enhances locally treated wound healing and does not function in a systemic manner.

### Key TRPA1 pathway mediators are induced in rosemary-treated ear wounds.

**Figure 5:**
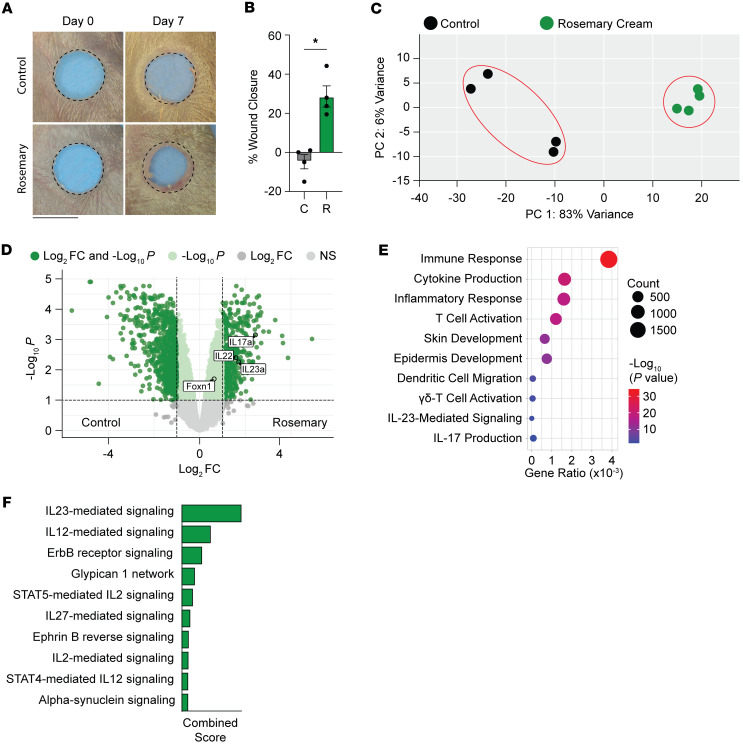
Transcriptome of rosemary cream-treated mouse ear wounds displays a TRPA1 pathway activation profile. (**A**) Representative photographs of vehicle control cream- or rosemary cream-treated WT ears at day 7 after injury. Dotted circle represents original 2 mm hole-punch size. Black scale bar represents 2 mm. (**B**) Percentage of wound closure at day 7 in vehicle control cream- (C; *n* = 4) or rosemary cream-treated (R; *n* = 4) WT mouse ears. Student’s *t* test 2-tailed and unpaired. *P < 0.05. Data are presented as mean ± SEM. (**C**) Principal component analysis of vehicle control cream- (black dots) or rosemary cream- (green dots) treated WT ears. (**D**) Volcano plot of all differentially expressed genes in WT mouse ears treated with vehicle control cream or rosemary cream. (**E**) Gene ontology analysis of biological processes significantly upregulated in rosemary cream-treated WT mouse ears. (**F**) Enrichr pathway analysis of WT mouse ears treated with rosemary cream. Combined score is computed by taking the log of the *P* value from the Fisher exact test and multiplying that by the *z* score of the deviation from the expected rank.

To further verify the involvement of the TRPA1-mediated signaling pathway in rosemary-mediated wound healing, we performed bulk RNA-Seq on wound edge tissue from ears treated for 1 week with rosemary or vehicle control cream (Figure 5, A and B). Principal component analysis demonstrated that treatment groups segregated across the first principal component, indicating that the biggest source of variation is between treatment groups (Figure 5C). Rosemary cream induced the expression of IL-23a, IL-22, and IL-17a transcripts, all components previously demonstrated to be necessary for TRPA1-mediated wound healing (Figure 5D). Additionally, gene ontology biological processes analysis revealed upregulation of γδ T cell activation, IL-23–mediated signaling, and IL-17 production. The Enrichr pathway with the highest combined score was IL-23–mediated signaling (Figure 5, E and F). Because of the observed decrease in fibrosis, we wanted to interrogate the pro-fibrotic myofibroblast response in rosemary-treated ears. We collected wound edge tissue of control cream- or rosemary cream-treated ears at 1 week after injury and assessed pro-fibrotic myofibroblast gene expression by qPCR. Compared with control cream, rosemary-treated ears exhibited 2-fold more *Acta2* expression and 2-fold less *Col3a1* expression (Supplemental Figure 3). We saw no differences in *Col1a1*, *Fn1*, and *Vim* levels (Supplemental Figure 3). We conclude that rosemary-treated ears may still induce myofibroblast generation and activation; however, the pro-regenerative pathway induced by TRPA1 may be more dominant than the fibrotic response. Taken together, these gene expression changes are aligned with our prior work dissecting how activation of TRPA1 receptor promotes scarless tissue regeneration.

### Neuron-specific TRPA1 deletion abrogates rosemary-induced wound healing.

**Figure 6:**
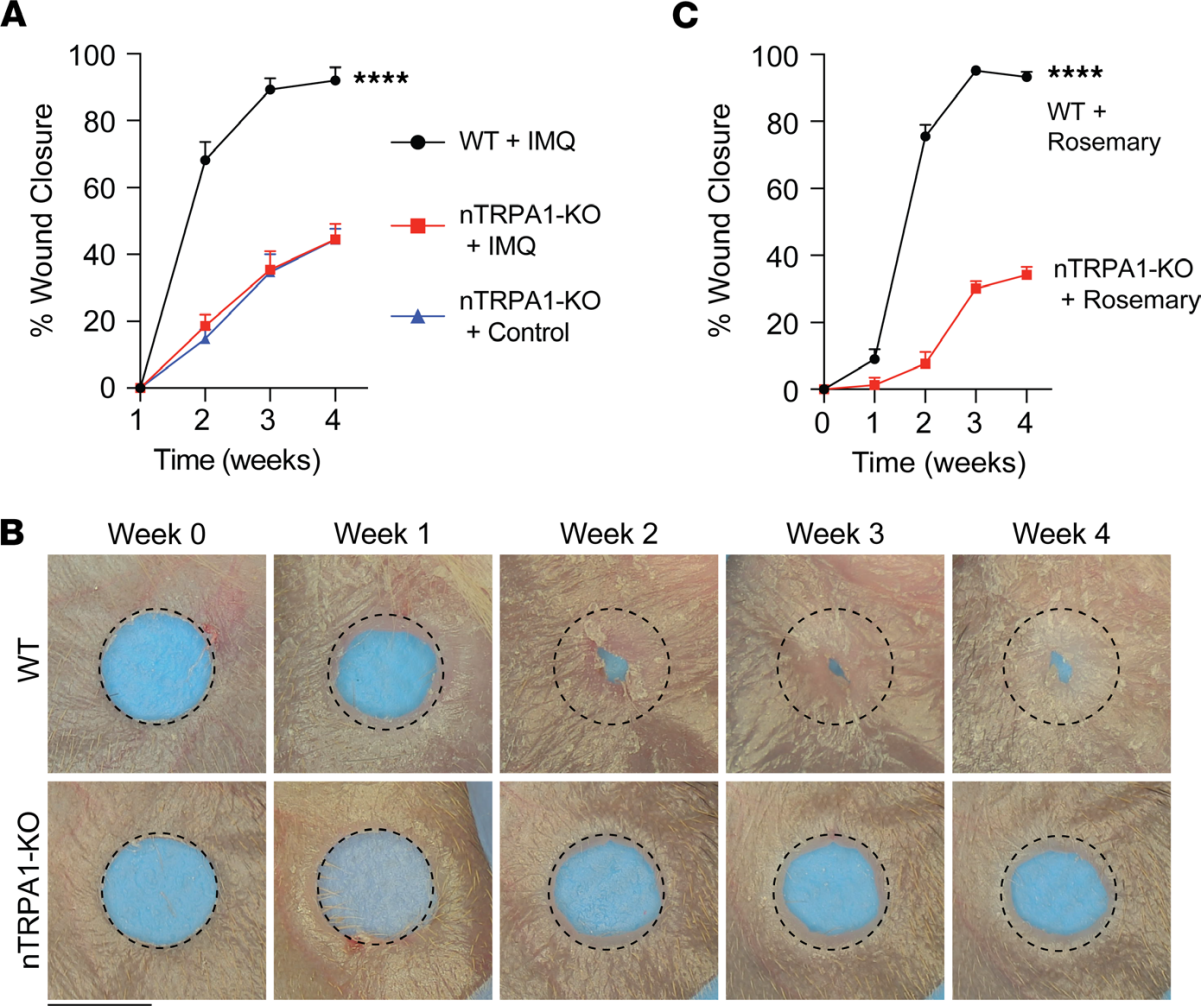
TRPA1 on cutaneous sensory neurons is necessary for rosemary cream-mediated ear wound regeneration. (**A**) Percentage of ear wound closure of WT littermate control mice treated with IMQ cream (solid black line, *n* = 6) or nTRPA1-KO mice treated with vehicle control cream (solid blue line, *n* = 3) or IMQ cream (solid red line, *n* = 5). Two-way ANOVA of WT + IMQ versus nTRPA1-KO + IMQ. (**B**) Representative photographs of rosemary cream-treated WT mouse ears or nTRPA1-KO mouse ears. Dotted circle represents original 2 mm hole punch size. Black scale bar represents 2 mm. (**C**) Percentage of ear wound closure of WT control mice treated with rosemary (solid black line, *n* = 8) or nTRPA1-KO mice treated with rosemary cream (solid red line, *n* = 12). Two-way ANOVA. Data are presented as mean ± SEM. ****P < 0.0001.

We and others have described that TRPA1 is mainly expressed in a subset of TRPV1-expressing neurons located in dorsal root ganglia (9, 25, 26). To test whether TRPA1-expressing neurons are necessary for rosemary-mediated tissue regeneration, we generated mice specifically lacking TRPA1 in sensory neurons (TRPA1^fl/fl^ TRPV1^Cre^, referred to as nTRPA1-KO). We previously demonstrated that daily application of topical IMQ promotes complete ear hole closure in WT mice (9). To confirm successful generation of nTRPA1-KO mice, we treated injured ears of nTRPA1-KO or WT littermate control mice with daily IMQ for 4 weeks. IMQ-treated WT mice closed their ear holes to a significantly smaller size compared with IMQ-treated nTRPA1-KO mice (~92% vs. ~45%, respectively, Figure 6A). Notably, vehicle-treated nTRPA1-KO mice also exhibited ~45% ear hole closure. Thus, neuron-specific TRPA1 is necessary for IMQ-induced tissue regeneration.

Next, we treated nTRPA1-KO mice with rosemary cream. We observed that rosemary cream-treated nTRPA1-KO mice closed their ear holes to a larger size compared with rosemary cream-treated WT control mice, thus exhibiting impaired tissue regeneration (Figure 6, B and C). Thus, peripheral nerve expression of TRPA1 is necessary for rosemary-mediated skin regeneration.

## Discussion

We demonstrate that topical rosemary extract enhances mammalian skin repair. Rosemary activates TRPA1^+^ sensory neurons to induce these effects, and carnosic acid within rosemary leaves acts as an active ingredient. Indeed, this work confirms that rosemary oils and cream, as reported on many social media sites, may reduce fibrosis and improve skin repair.

Plant extracts have long been used in traditional medicine, but oftentimes the active ingredient remains unknown or unidentifiable. We successfully identified that carnosic acid in rosemary leaves is a key biologically active component that promotes scarless tissue regeneration. Our plant screen also identified oregano and thyme as other plants that activate TRPA1, and prior work demonstrated that both plants generate carnosic acid or carnosol, an oxidation product of carnosic acid (19, 27). Both products are reported to activate TRPA1 (20). TRPA1 receptor activation is primarily triggered by electrophilic irritants such as AITC (mustard oil), cinnamaldehyde, or JT010. Future studies are needed to assess whether oxidation of carnosic acid to carnosol is necessary to promote scarless wound healing. We identified that mint, fennel, nutmeg, and allspice may also activate TRPA1. We speculate that these plants may also be compounded into a cream to promote wound healing. However, more work is needed to determine their active ingredient(s).

Rosemary extract exhibited minor activation of the TRPV3 receptor. We did not detect any notable TRPV3 agonists in our UPLC/MS analysis. Prior literature demonstrated that carnosol and carnosic acid do not activate TRPV3, and TRPV3 activation has not been reported to induce wound healing (20). Thus, we do not believe that TRPV3 activation is a contributing factor in our studies.

Human and mouse skin exhibit many differences, including resident immune cell populations. We previously showed that TRPA1-mediated tissue regeneration requires γδ T cells, which may reside in the epidermis or dermis. While mice have more γδ T cells in the epidermis (also known as dendritic epidermal T cells) compared with humans, both mouse and human skin contain similar numbers of dermal γδ T cells. Moreover, mouse and human dermal γδ T cells were shown to promote wound healing via secreting insulin-like growth factor 1 and fibroblast growth factor 9 (28–31). We speculate that TRPA1 activation may promote tissue regeneration through either population of γδ T cells, and rosemary extract may induce scarless tissue regeneration in human skin. While CD4^+^ helper T cells have been reported to express TRPA1, they express TRPA1 by orders of magnitude lower than sensory neurons, and TRPA1 expression in CD4^+^ helper T cells was shown not to be a key regulator of T cell receptor–stimulated calcium signaling (32). Additionally, the calcium influx of TRPA1 in these cells has not been reported to mediate wound healing. Taken together, we think that T cells contribute to the enhanced wound healing, but it is not a direct effect via TRPA1 on T cells. Additional studies are needed to explore these possibilities.

While IMQ and AITC have proven effective to promote scarless wound healing, potential side effects have limited their use in regenerative medicine (33–35). IMQ was originally designed to activate TLR7 to promote inflammation and innate immunity, and its ability to activate TRPA1 was a nonspecific side effect. AITC is the gold-standard TRPA1 agonist and is a well-established caustic chemical that may cause severe chemical-induced irritation or burns to tissues. Our rosemary extract is substantially less caustic than AITC. Moreover, carnosic acid has not been reported to activate the TLR7 pathway or to possess corrosive or tissue-damaging properties. In fact, carnosic acid may attenuate allergic inflammatory mediators in activated mast cells and function as an antioxidant (19, 36, 37). Carnosic acid is typically localized within the chloroplasts, the organelles in plant leaves responsible for photosynthesis. It acts as a free radical scavenger and neutralizes harmful reactive oxygen species generated during photosynthesis (19, 38). Being less caustic may come at a cost. Our prior work showed that activation of TRPA1 could induce a systemic wound healing effect. We observed that activation of TRPA1 by rosemary did not induce a systemic wound healing effect. We believe this may be related to the concentration of agonist in these experiments. Rosemary cream was approximately 40% as effective as AITC in our in vitro experiments (Figure 4). This difference may also explain why AITC is more caustic than rosemary cream. Further studies will be needed to determine optimal concentrations of rosemary for local and systemic wound healing effects. Taken together, rosemary extract or carnosic acid may represent an ideal TRPA1 agonist to promote local scarless tissue regeneration.

These results demonstrate that the enthusiasm publicized on social media may be correct, and rosemary-containing extracts reduce mammalian skin scar formation. These results coupled with the widespread accessibility of the rosemary plant and its relatively low cost should motivate formal testing of rosemary-containing creams to reduce scar formation in human clinical trials.

## Methods

### Sex as a biological variable.

This wound healing study examined male and female animals; similar findings are reported for both sexes. Further studies are needed to assess whether the rosemary plant can display sex-specific effects.

### Animal models.

C57BL/6J (strain 000664), 129S-*Trpa1^tm2Kykw^*/J (TRPA1^fl/fl^, strain 008649), and B6.129-*Trpv1^tm1(cre)Bbm^*/J (TRPV1^Cre^, strain 017769) mice were obtained from The Jackson Laboratory. TRPA1^fl/fl^ mice were crossed with TRPV1^Cre^ mice to obtain mice deficient for TRPA1 specifically in TRPV1-expressing neuronal cells. All mouse genotypes were verified by standard PCR as described by The Jackson Laboratory (protocols 28644 and 35042). Mice were housed at the University of Pennsylvania animal care facilities on a 12-hour light/12-hour dark cycle with free access to normal chow and water. All mice were used between 6 and 8 weeks of age.

### Animal injury.

For ear wounding, we used a standard 2 mm mechanical punch (Roboz) to create a through-and-through hole in the center of each outer ear (pinna). We applied ~50 mg of treatment cream to injured mouse ears every day for 1 month. Ear hole diameter of wound edge tissue was measured using a dissection microscope (Nikon) in the horizontal and vertical directions on a weekly basis. Preestablished criteria required the exclusion of ears if there were signs of wound infection, tearing of the ear, or abnormal geometric shape. No points were excluded in this study. For WIHN, 1.5 cm^2^ full-thickness skin wounds were made as previously described (24). We applied ~50 mg of treatment cream adjacent to the wounded area every day for 4 weeks. After 4 weeks, de novo hair follicles were identified by whole-mount alkaline phosphatase staining of dermis preparations as previously described (24). For stented back wounds, a 6 mm disposable biopsy punch (Acuderm) was used to make 2 circular full-thickness wounds on the dorsal back skin of mice. Silicon wound splints (Grace Bio-Labs) were sutured with 4-0 nylon to prevent skin contracture. We applied ~50 mg of treatment cream around the stented wound every day for 4 weeks. Reepithelialized skin borders were used to measure percentage wound closure.

### Injury treatments.

To generate the rosemary and cilantro creams, dried rosemary or cilantro extract was resuspended in 500 μL of DMSO. Cetaphil cream was added to obtain a final concentration of 12 mg of rosemary or cilantro extract/mL. Carnosic acid cream was generated by resuspending 50 mg of carnosic acid (Santa Cruz Biotechnology, sc-202520A) in 250 μL DMSO. Cetaphil cream was added to obtain a final concentration of 5 mg/mL. Vehicle control cream for each treatment was generated by adding an equal volume of treatment vehicle to Cetaphil cream. IMQ cream USP 5% (12.5 mg/0.25 g, NDC 68462-536-70) was obtained from Glenmark Pharmaceuticals.

### Plant extractions.

Fresh rosemary or cilantro plant leaves (Trader Joe’s) were weighed (~15 g) and minced in the presence of ethanol (~30 mL) in a 50 mL conical. The minced leaves in ethanol were stored overnight at –80°C. The minced leaves were then discarded, and the liquid solution was filtered through a 0.22 μm vacuum filtration system (03359, Sigma). The ethanol in the solution was evaporated under vacuum in a 37°C water bath to obtain the dried rosemary or cilantro extract. For TRP calcium flux assays, fresh herb/plant leaves or spices (200 mg) were minced in DMSO and extracted overnight at 4°C, clarified by centrifugation at 10,000*g* for 10 minutes, and stored at –20°C. The DMSO extract was diluted to 0.25 mg/mL in LHC-9 (12680013, Gibco) for calcium flux assays.

### UPLC/MS analysis.

Chromatographic separation was performed on a Waters Acquity UPLC I-Class Plus Instrument equipped with a binary gradient pump and TUV 2 channel UV detector. A Waters Acquity UPLC HSS C18 column (2.1 × 50 mm, 1.8 μm particle size), along with a prefilter, was used with the column temperature maintained at 30°C. A binary eluent system was employed with mobile phase A (water with 0.1% formic acid) and mobile phase B (acetonitrile with 0.1% formic acid) with a flow rate of 0.5 mL/min and a gradient program as follows: 0.00–0.50 minutes, 5% B; 0.50–12.50 minutes, linear from 5% to 95% B; 12.50–14.50 minutes, 95% B; 14.50–15.00 minutes, 5% B. For the preparation of the eluents, Optima LC/MS-grade water, acetonitrile, and formic acid were purchased from Thermo Fisher Scientific and used without further purification. The sample injection volume was 2 μL. The UV detector was set to 254 nm.

The UPLC instrument was coupled to a Waters SQD2 single quadrupole mass spectrometer with a Zspray electrospray ionization (ESI) source using nitrogen as desolvation and nebulization gas. Source conditions were as follows: capillary voltage, 1.50 kV; cone voltage, 30 V; desolvation temperature, 600°C; desolvation gas flow rate, 1,000 L/h; and cone gas flow rate, 0 L/h. The mass spectrometer was operated in negative ESI-mode scanning *m/z* 50–1,000.

### TRP channel overexpression.

Human TRPA1, V1, V2, V3, V4, and M8 genes were subcloned into the pcDNA 3.1D-V5/His6 mammalian expression vector (Invitrogen), as previously described (39–41). Individual plasmids were transfected into HEK293 cells (ATCC), which exhibit minimal/absent basal response to the TRP channel agonists. Stable lines were selected by using Geneticin (400 μg/mL) (Invitrogen) and isolating homogeneous colonies by dilution. TRPM2-overexpressing HEK293 cells were provided by Yasuo Mori (Kyoto University, Kyoto, Japan) and generated as previously described (42, 43). All overexpressing cells were maintained in DMEM/F12 supplemented with 5% FBS, 1× antibiotic and antimycotic, and 300 μg/mL Geneticin.

### TRP screening assays/calcium influx assays.

Calcium flux assays were performed as previously described (44). Briefly, HEK293 cells stably overexpressing TRPA1, TRPM2, TRPM8, TRPV1, TRPV2, TRPV3, or TRPV4 were used. Changes in fluorescence intensity indicative of an increase in intracellular calcium were measured using the Fluo-4 Direct assay kit (F10472, Thermo Fisher Scientific) on a BMG Labtech NOVOStar fluorescence plate reader. TRP-overexpressing HEK293 cells were plated in flat-bottom, 96-well plates coated with 1% gelatin at about 30,000 cells/well and assayed at 90% confluence 1–2 days postplating. Prior to the assay, the cells were loaded with 1× Fluo-4 diluted 1:1 in LHC-9 at 37°C for 1 hour. The Fluo-4 was removed, and the cells were washed with LHC-9 containing 1 mM probenecid (P36400, Thermo Fisher Scientific) and 0.75 mM trypan red (2456, ATT Bioquest). The assays were performed at 37°C. Plant extract treatments (0.25 mg/mL in DMSO) were added to cells at 3 times the desired final concentration in LHC-9 (111.1 μM calcium). Reported values were corrected by subtracting the fluorescence response to a negative media control (i.e., no agonist) containing the appropriate concentrations of DMSO to match the extract samples. The data were further corrected by subtracting the fluorescence response of HEK293 cells lacking TRP expression with the data reported as the percentage of response relative to each TRP channel’s respective specific agonist (positive control): TRPA1 = 250 mM AITC, TRPM2 = 1 mM H_2_O_2_, TRPM8 = 50 mM icilin, TRPV1 = 1 mM nonivamide, TRPV2 = 250 mM D^9^-tetrahydrocannabinol, TRPV3 = 250 mM carvacrol, or TRPV4 = 35 nM GSK 1016790A.

### Histology.

Standard histology and immunostaining protocols were followed, and tissue origin was blinded during histologic staining. In brief, the fresh skin tissue was fixed overnight at 4°C in 4% paraformaldehyde (J19943-K2, Thermo Fisher Scientific). The tissue was dried in 70% ethanol, trimmed, placed into tissue cassettes, processed (VIP5b, Sakura), and embedded into wax (Leica Paraplast X-tra) blocks. Blocks were cut using disposable blades (D554P, Sturkey) on a rotary microtome (RM2235, Leica) set at 5 μm thickness. Sections were floated on a water bath (145702, Boekel) set at 43°C and collected onto positively charged glass slides (Fisherbrand Superfrost Plus, Thermo Fisher Scientific). Following overnight drying at room temperature, slides were baked for 30 minutes at 60°C, followed by H&E staining using an automated stainer (Leica Autostainer XL). Tissue embedding, sectioning, staining, and slide processing were performed in the Skin Biology and Disease Resource–Based Core at the Department of Dermatology, University of Pennsylvania. H&E-stained sections were examined under bright-field microscopy and images acquired on a Keyence BZ-X700 microscope.

### PSR staining.

The paraffin sections were dewaxed and hydrated, and the nuclei were stained with hematoxylin. PSR (Polysciences Inc., NC9908782) was added for 1 hour. The slides were washed twice with acidified water (0.05% glacial acetic acid). The slides underwent dehydration in 3 changes of 100% ethanol, followed by clearing in xylene, and finally, mounting in a resinous medium. The PSR images were acquired on a Leica DM6B-Z microscope using a light polarizer equipped with a 32 mm quarter-wave plate and an ICT/P analyzer module. The acquired images were analyzed for fibrosis by quantifying the percentage of collagen 1 (PSR) signal within the wounded tissue region using FIJI. Representative images were selected for figure panels.

### RNA isolation.

A 6 mm circular biopsy punch was used to capture the tissue surrounding the 2 mm ear wound. The peripheral wound tissue from vehicle control cream-treated and rosemary cream-treated mouse ear wounds was homogenized using a Tissue-Tearor (985370, BioSpec Products), and RNA was isolated using an RNeasy kit (74106, QIAGEN).

### Real-time RT-PCR.

Total RNA was quantified by UV absorbance on a NanoDrop (Thermo Fisher Scientific), and cDNA (2 μg) was synthesized using the QuantiTect Reverse Transcription Kit (205313, QIAGEN). The cDNA was then subjected to analysis by qPCR using TaqMan Fast Gene Expression Master Mix (Thermo Fisher Scientific) and a Life Technologies QuantStudio 7 Pro instrument. The following TaqMan probe-based assays were used: Actb (Mm02619580_g1), Acta2 (Mm00725412_s1), Vim (Mm01333430_m1), Col1a1 (Mm00801666_g1), Col3a1 (Mm00802300_m1), and Fn1 (Mm01256744_m1. mRNA expression was normalized to Actb and reported as relative expression using the comparative ΔΔCt method.

### Bulk RNA library preparation.

The RNA-Seq libraries were made using the NEBNext Ultra II DNA Library Prep Kit for Illumina (E7760S, New England Biolabs) and NEBNext Multiplex Oligos for Illumina (E6609S, New England Biolabs) following the manufacturer’s instructions and sequenced using 2 × 100 bp paired-end runs on Illumina HiSeq 2000/HiSeq 2500 platforms at BGI Americas.

### Bulk RNA-Seq analysis.

FASTQ files were aligned to the GRCm39 reference genome using STAR_2.4.0 in basic 2-pass mode using the Encode options as specified in the manual. Resulting BAM files were turned into a count matrix using the featureCounts program. Normalization by fragments per kilobase per million mapped fragments counts and differentially expressed transcripts were generated using the R packages edgeR and Limma, respectively.

### Statistics.

For in vivo time courses comparing hole size, data were analyzed using 2-way ANOVA using a temporal main effect, a main effect comparing treatment, and an interaction of the 2 main effects. For tests such that the 2-way ANOVA indicates significant time-treatment interactions, an additional 2-tailed Student’s *t* test was used, with *P* values of less than 0.05 considered significant. For all other analyses, comparisons between 2 groups were carried out using a 2-tailed unpaired Student’s *t* test unless otherwise indicated in the figure legend. In all tests, a *P* value of less than 0.05 is considered significant.

### Study approval.

Experiments involving mice were reviewed and approved by the Institutional Animal Care and Use Committee of the University of Pennsylvania (protocol #805620). Mice were treated in accordance with the NIH *Guide for the Care and Use of Laboratory Animals* (National Academies Press, 2011).

### Data availability.

The raw RNA-Seq data files can be accessed with GEO accession number GSE298490 at https://www.ncbi.nlm.nih.gov/geo/query/acc.cgi?acc=GSE298490 This paper does not report any original code. Any additional information required to reanalyze the data reported in this paper is available from the lead contact upon request.

The Supporting Data Values file is provided in the supplement.

## Author contributions

Experiment conceptualization, methodology, and investigation were performed by ER, JP, BS, SMP, CAR, and THL. Bioinformatics analysis was performed by ER and JP. Writing of the manuscript was performed by ER, JP, and THL. Reviewing and editing the manuscript were performed by CAR and THL. The order of authors in the first position of the author byline was determined by prioritizing manuscript writing contribution. These criteria were preestablished and agreed upon by all authors.

## Funding support

This work is the result of NIH funding, in whole or in part, and is subject to the NIH Public Access Policy. Through acceptance of this federal funding, the NIH has been given a right to make the work publicly available in PubMed Central.

THL from Berstein Family, NIH (R01AR079483), VA (I01RX002701), Edwin and Fanny Gray Hall Center for Human Appearance, and H.T. Leung Foundation.

## Supplementary Material

Supplemental data

Supporting data values

## Figures and Tables

**Table 1:**
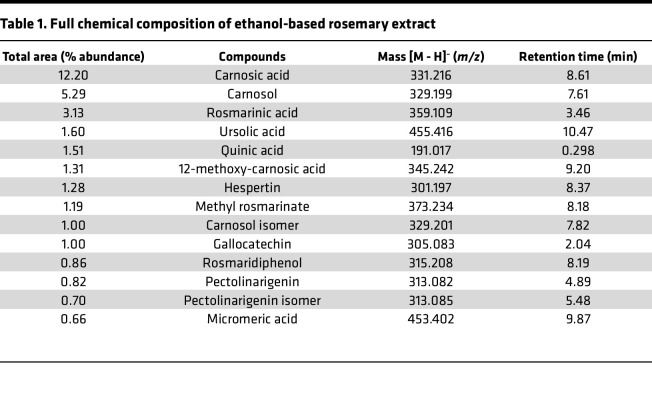
Full chemical composition of ethanol-based rosemary extract

## Version 1 10/23/2025

In-Press Preview

## Version 2 12/08/2025

Electronic publication
