# Supplementary material for: Carnosic acid in topical rosemary extract enhances skin repair via TRPA1 activation
Source: JCI Insight. 2025 Oct 23;10(23):e196267. doi: 10.1172/jci.insight.196267 (PMC12890508; doi:10.1172/jci.insight.196267)
Supplement: Supplemental data [file jciinsight-10-196267-s068.pdf]

A

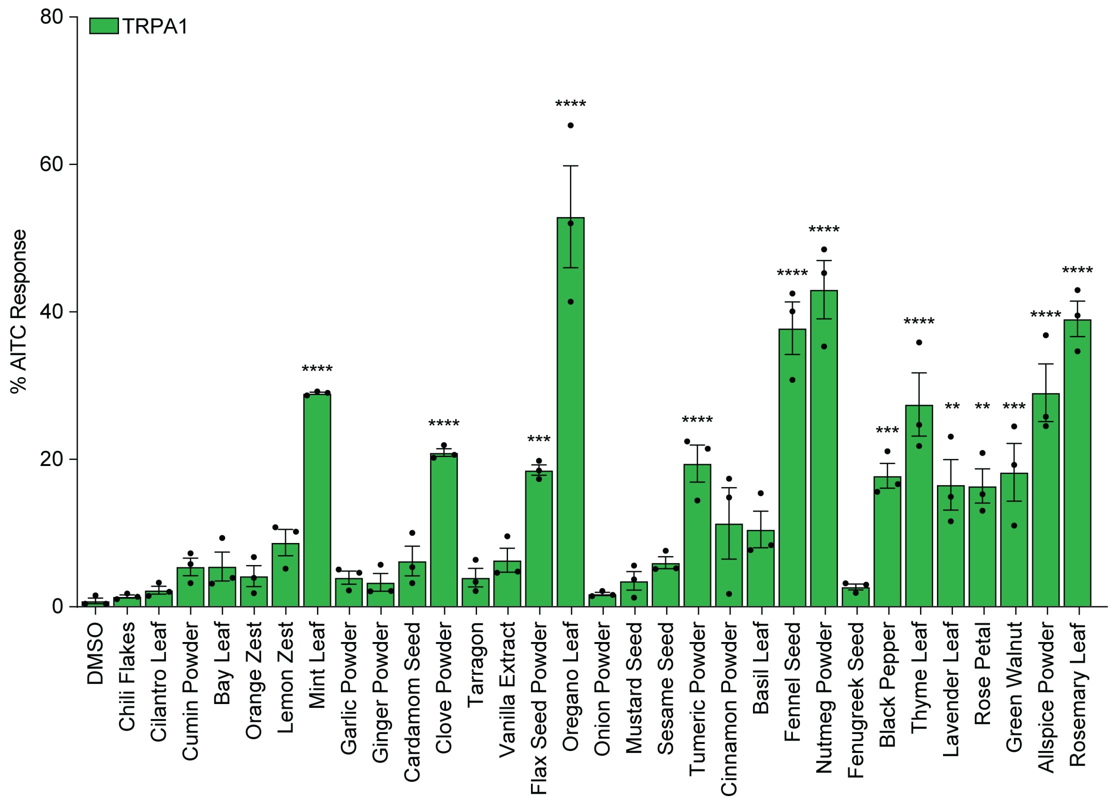

Supplemental Figure 1: Rosemary, among other herbs, activates TRPA1.

(**A**) Calcium flux assay responses of TRPA1 over-expressing HEK cells treated with different extracts reported as a percent of the TRPA1 specific agonist, AITC. One-way ANOVA for extract response compared to DMSO control response. Data are presented as mean ± SEM. *$P<0.05$, **$P<0.01$, ***$P<0.001$, ****$P<0.0001$.

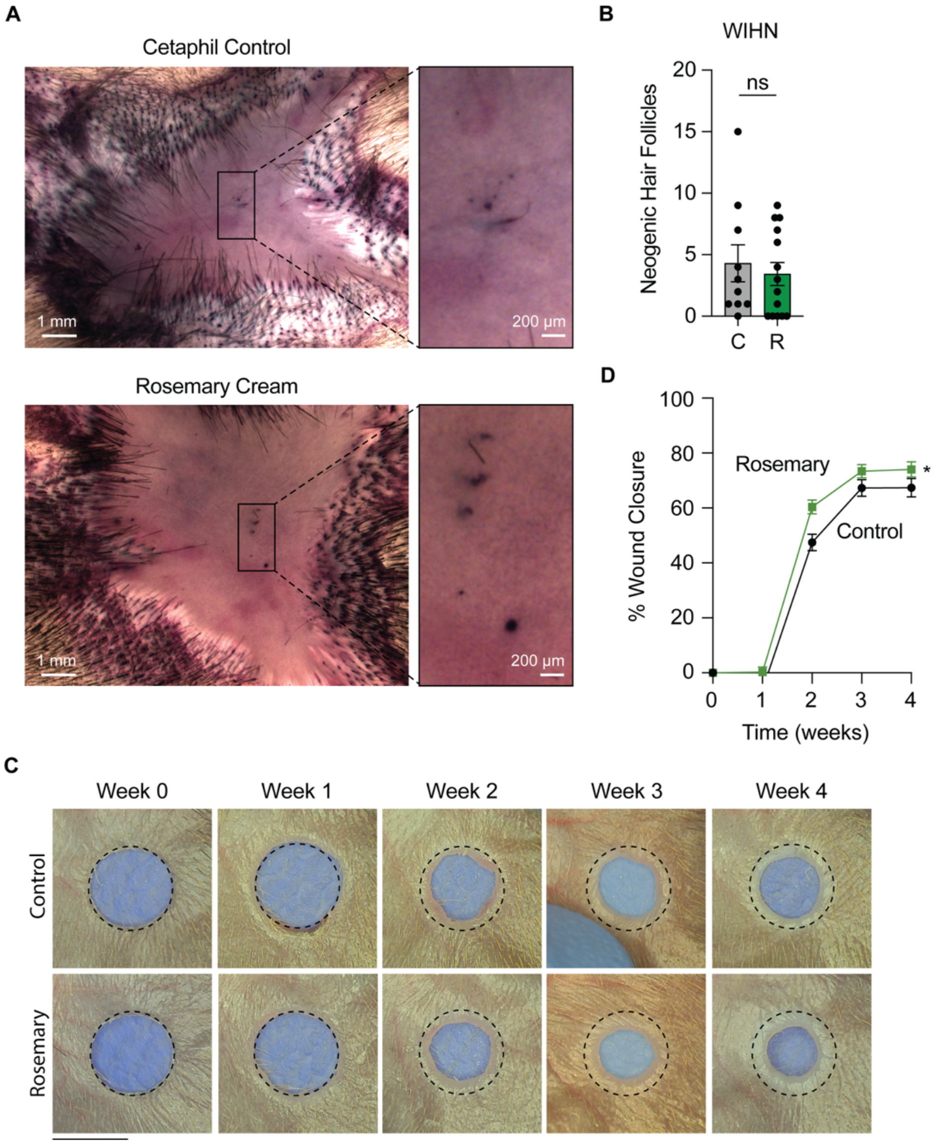

Supplemental Figure 2: Rosemary does not promote systemic wound healing.

(**A**) Representative photographs of hair follicle regeneration in WIHN from WT mice whose ears were treated with vehicle control cream or rosemary cream. (**B**) Quantification of hair follicle regeneration in WIHN from WT mice whose ears were rubbed with vehicle control cream (n=10) or rosemary cream (n=14). Student's t-test two tailed and unpaired. (**C**) Representative photographs of WT mouse ears whose backs were treated with vehicle control cream or rosemary cream. Dotted circle represents original 2mm hole-punch size. Black scale bar represents 2 mm. (**D**) Percentage of ear wound closure in WT mice whose backs were treated with vehicle control cream (solid black line, n=20) or rosemary cream (solid green line, n=22). Two-way ANOVA. Data are presented as mean ± SEM. *P<0.05, **P<0.01, ***P<0.001, ****P<0.0001.

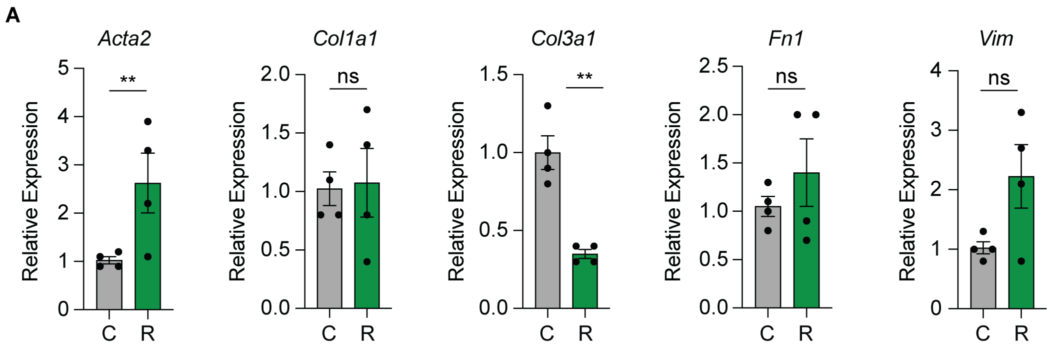

Supplemental Figure 3: Rosemary-treated ears may still induce myofibroblast activation.

(**A**) Relative expression of *Acta2, Col1a1, Col3a1, Fn1,* and *Vim* measured by qPCR in injured ears treated with vehicle control cream or rosemary cream (C = control, R = rosemary). Student's t-test two tailed and unpaired. Data are presented as mean ± SEM. *P<0.05, **P<0.01, ***P<0.001, ****P<0.0001.
